# Compartmentalization of Immune Response and Microbial Translocation in Decompensated Cirrhosis

**DOI:** 10.3389/fimmu.2019.00069

**Published:** 2019-02-08

**Authors:** Camila Alvarez-Silva, Robert Schierwagen, Alessandra Pohlmann, Fernando Magdaleno, Frank E. Uschner, Patrick Ryan, Maria J. G. T. Vehreschild, Joan Claria, Eicke Latz, Benjamin Lelouvier, Manimozhiyan Arumugam, Jonel Trebicka

**Affiliations:** ^1^Novo Nordisk Foundation Center for Basic Metabolic Research, Faculty of Health and Medical Sciences, University of Copenhagen, Copenhagen, Denmark; ^2^Department of Internal Medicine I, Goethe University Clinic Frankfurt, Frankfurt, Germany; ^3^Department of Internal Medicine I, University of Bonn, Bonn, Germany; ^4^Medical Clinic II, University Center for Infectious Diseases, University Hospital Frankfurt, Frankfurt, Germany; ^5^European Foundation for the Study of Chronic Liver Failure, Barcelona, Spain; ^6^Institute of Innate Immunity, University of Bonn, Bonn, Germany; ^7^Division of Infectious Diseases and Immunology, University of Massachusetts Medical School, Worcester, MA, United States; ^8^German Center for Neurodegenerative Diseases (DZNE), Bonn, Germany; ^9^Centre for Inflammation Research, Norwegian University of Science and Technology, Trondheim, Norway; ^10^Vaiomer, Labège, France; ^11^Department of Gastroenterology and Hepatology, Odense University Hospital, Odense, Denmark; ^12^Institute for Bioengineering of Catalonia, Barcelona, Spain

**Keywords:** microbiome, systemic inflammation, myeloid cells, cirrhosis, ascites, cytokines, acute-on-chronic liver failure

## Abstract

**Background:** Acquired dysfunctional immunity in cirrhosis predisposes patients to frequent bacterial infections, especially spontaneous bacterial peritonitis (SBP), leading to systemic inflammation that is associated with poor outcome. But systemic inflammation can also be found in the absence of a confirmed infection. Detection of bacterial DNA has been investigated as a marker of SBP and as a predictor of prognosis. Data is, however, contradictory. Here we investigated whether levels of IL-6 and IL-8 putatively produced by myeloid cells in ascites are associated with systemic inflammation and whether inflammation depends on the presence of specific bacterial DNA.

**Methods and Materials:** We enrolled 33 patients with decompensated liver cirrhosis from whom we collected paired samples of blood and ascites. IL-6 and IL-8 were measured in serum samples of all patients using ELISA. In a subset of 10 representative patients, bacterial DNA was extracted from ascites and whole blood, followed by 16S rRNA gene amplicon sequencing.

**Results:** There were significantly higher levels of IL-6 in ascites fluid compared to blood samples in all patients. Interestingly, IL-6 levels in blood correlated tightly with disease severity and surrogates of systemic inflammation, while IL-6 levels in ascites did not. Moreover, patients with higher blood CRP levels showed greater SBP prevalence compared to patients with lower levels, despite similar positive culture results. Bacterial richness was also significantly higher in ascites compared to the corresponding patient blood. We identified differences in microbial composition and diversity between ascites and blood, but no tight relationship with surrogates of systemic inflammation could be observed.

**Discussion:** In decompensated cirrhosis, markers of systemic inflammation and microbiota composition seem to be dysregulated in ascites and blood. While a relationship between systemic inflammation and microbiota composition seems to exist in blood, this is not the case for ascites in our hands. These data may suggest compartmentalization of the immune response and interaction of the latter with the microbiota especially in the blood compartment.

## Introduction

Acquired dysfunctional immunity in cirrhosis predisposes patients to frequent bacterial infections contributing to disease progression and may lead to the development of acute-on-chronic liver failure (ACLF) (1). Especially, spontaneous bacterial peritonitis (SBP) is one of the most frequent infections in cirrhosis and therefore a trigger for ACLF (2). Interestingly, SBP is defined by the number of granulocytes in ascites and not by cultural detection of bacteria (3). This is because many patients with SBP show negative bacterial cultivation from ascites, while in many other patients, viable bacteria can be cultivated in the absence of peritonitis, so-called bacterascites (3). Lack of bacterial cultivation in the presence of SBP can be explained by the concept of dormant microbiota, meaning that bacteria can reside inside circulating and resident immune cells but cannot be cultured and hence detected (4).

The source of infection, especially in the case of SBP, is suspected to be bacterial translocation from the gut (2), which is facilitated by impaired intestinal defense and increased intestinal permeability, processes which are correlated with the stage of cirrhosis (5). Not only viable bacteria (1, 6), but also bacterial components broadly termed pathogen-associated molecular patterns (PAMPs) may induce an immune response and aggravate systemic inflammation (2). As a consequence, the presence of circulating bacterial DNA reflects bacterial translocation in cirrhosis. However, the relationship of bacterial DNA with systemic inflammation is still controversially discussed (7, 8). PAMP-associated immune response requires recognition by innate immune cells and is reflected by synthesis and secretion of cytokines. Especially levels of the cytokines IL-6 and IL-8 are closely related to ACLF and clinical outcome (9). At the same time, the antibacterial capacity of myeloid cells and myelopoiesis is impaired during decompensation and ACLF (10, 11). Interestingly, these effects are not only induced by PAMPs (deriving from pathogens), but in general by bacterial-derived molecules, so-called microbe-associated molecular patterns (MAMPs).

Recent data from our group suggest that specific circulating bacteria or MAMPs in blood are associated with myeloid cell dysregulation and systemic inflammation (12). Therefore, a specific microbial DNA may be more crucial than just detecting any bacterial DNA in circulation. In ascites, the detection of bacterial DNA in ascites does not seem to correlate with SBP or clinical signs of infection (13). Therefore, the question remains, whether specific bacteria in ascites lead to myeloid cell activation with subsequent cytokine release and a systemic inflammatory response. To address this question, we investigated whether the representative cytokine (IL-6, IL-8) levels produced by myeloid cells in ascites reflect systemic inflammation. Moreover, we compared the levels of cytokines in paired ascites and blood samples, together with the general bacterial composition and specific species abundances of the microbiota estimated by 16S ribosomal RNA gene sequencing. Our study focused not only on common pathogens but also on the role of MAMPs in activating myeloid cells of the innate immune response. We hypothesize that DNA from specific bacterial species, but not any bacterial DNA, induces an inflammatory response in cirrhotic patients.

## Materials and Methods

### Patient Recruitment and Sample Collection

Ascites and blood were collected in 33 cirrhotic patients scheduled for paracentesis. Paracentesis was performed by international standards (14). The study was performed in accordance with the declaration of Helsinki and approved by the local ethics committee at the University of Bonn (Nr. 121/14). Patients gave their written informed consent.

### Microbiological Analysis

Microbiological culture of blood and ascites was performed at the clinical microbiology department at the University of Bonn in accordance with Microbiology Procedures Quality Standards of the German Society for Hygiene and Microbiology (15, 16). Ten milliliters of peripheral whole blood or ascitic fluid were transferred into aerobic, anaerobic and fungal blood culture bottles and incubated for up to 5 days using the Bactex FX blood culture system as described previously (17). SBP was diagnosed based on a leucocyte count in ascites >250/μl (3).

### Enzyme-Linked Immunosorbent Assay

Ascites and peripheral blood samples (serum, EDTA-plasma and whole-blood) were collected during paracentesis and stored immediately at −80°C until further use. Enzyme-linked immunosorbent assay for cytokines IL-6 and IL-8 (DY206 and DY208, respectively; R&D Systems Inc., Minneapolis, MN, USA) were performed according to the manufacturer's protocols. All samples were measured in duplicates.

### 16S Ribosomal RNA Gene Sequencing

For extraction of total DNA, 100 μl of whole blood or ascitic fluid were used. Samples were mechanically lysed two times for 30 s at 20 Hz in a bead beater (TissueLyser, Qiagen, Hilden, Germany) with 0.1 mm glass beads (MoBio, CA, USA). DNA extraction was performed using the NucleoSpin Blood Kit (Macherey-Nagel, Düren, Germany). Quality and quantity of extracted nucleic acids were controlled by gel electrophoresis. 16S rRNA gene V3-V4 hypervariable regions were amplified and quantified by qPCR, and sequenced using MiSeq technology (Illumina, San Diego, CA, USA) as described previously producing 2 × 300 bp paired-end reads (18, 19).

### Bioinformatics and Statistical Analysis

Paired-ends reads were processed in DADA2 version 1.6.0 (20) using the following parameters: “trimLeft = 10, truncLen = (260;230), maxN = 0, maxEE = (2,5), minOverlap = 20, maxMismatch = 1, pool = TRUE.” The resulting output was an amplicon sequence variant (ASV) table. The ASVs were mapped against SILVA database (21) release 128. Data analysis was performed in R version 3.5, using phyloseq package (22) version 1.24.2. Genus level relative abundances were generated by combining ASVs annotated with the same genus using phyloseq package. The alpha-diversity measures were calculated based on rarefied data at 6,000 reads/sample. Significant differences in alpha-diversity between ascites and blood samples as well as between samples with different inflammation status were determined using *t*-test.

Beta-diversity was calculated using unweighted and weighted UniFrac measures. Effects of different factors on beta-diversity were determined using Adonis Permutational Multivariate Analysis of Variance (PERMANOVA) test from the vegan package (23) version 2.5.2. For differential abundance analysis, we chose a *t*-test based on the evaluation using DAtest package (24) version 2.7.11, which showed that, compared with different statistical tests, *t*-test has the highest area under the curve (AUC = 0.608) while keeping the false discovery rate (FDR) under 0.05.

Statistical analysis of patient characteristics and clinical measures was performed using SPSS version 24 (IBM, Armonk, NY) and Mann–Whitney test for metric variables and Chi-square test for nominal variables. Receiver operator characteristic curve analysis was used to define a cut-off for CRP and divide the cohort into one group with low CRP profile and one group with high CRP profile. *P*-values were adjusted for multiple testing using Benjamini–Hochberg FDR procedure. An FDR-adjusted *P*-value below 0.05 was considered statistically significant.

## Results

### General Description of the Cohort

General characteristics of the patients are summarized in Table 1. The cohort consists of patients with decompensated liver cirrhosis as reflected by the model for end-stage liver disease (MELD) and Child-Pugh (CHILD) scores, which assess severity of liver disease, prognosis and mortality. The median MELD score of 15 represents a value at which patients would benefit from liver transplantation. The median CHILD score of 10 is associated with a 1-year survival below 50%. Seventy-two percent of the patients (24/33) showed ACLF at baseline.

**Table 1:** Patient characteristics.

	**Parameters**	**All (*n* = 33)**	**Low CRP profile (*n* = 12)**	**High CRP profile (*n* = 21)**	***P***
General characteristics	Sex (male/female)	23/10	8/4	15/6	0.78
	Age, years	57 (33–105)	53.5 (40.0–75.0)	60.0 (33.0–105.0)	0.19
	Etiology of cirrhosis (alcohol/viral/other)	18/8/7	8/4/0	10/4/7	0.08
	MELD	15.71 (3.66–39.65)	13.87 (9.79–38.25)	20.50 (3.66–39.65)	0.06
	CHILD	10 (7–15)	9 (7–13)	10 (7–15)	**0.03**
	ACLF (no/yes)	9/24	5/7	4/17	0.16
	CLIF-C-ACLF	57 (39–84)	44 (39–57)	60 (47–84)	**0.002**
	Esophageal varices (absent/small/large)	14/8/11	6/2/4	8/6/7	0.38
	Hepatic encephalopathy (absent/grade 1/grade 2/grade 3/grade 4)	22/4/3/2/2	11/1/0/0/0	11/3/3/2/2	0.20
	Hepatorenal syndrome (no/yes)	14/19	6/6	8/13	0.51
	Spontaneous bacterial peritonitis (no/yes)	26/7	12/0	14/7	**0.02**
	Blood culture (negative/positive/NA)	12/6/15	4/2/6	8/4/9	1.00
	Ascites culture (negative/positive/NA)	24/6/3	11/1/0	13/5/3	0.19
Laboratory blood	Creatinine [mg/dl]	1.40 (0.57–4.68)	1.30 (0.57–2.59)	1.58 (0.86–4.68)	0.10
	Platelets [10^6^/l]	120 (7–685)	108 (38–235)	120 (7–685)	0.84
	INR	1.4 (1.0–5.5)	1.35 (1.00–2.10)	1.40 (1.00–5.50)	0.78
	Bilirubin [mg/dl]	2.08 (0.37–45.21)	1.92 (0.75–45.21)	3.20 (0.37–35.41)	0.81
	GGT [U/l]	152 (26–1,139)	139 (33–585)	181 (26–1,139)	0.76
	ALT [U/l]	32 (8–1,050)	49.5 (17.0–269.0)	30.0 (8.0–1050.0)	0.75
	CRP [mg/l]	48.8 (5.7–205.0)	13.55 (5.70–22.20)	60.20 (27.60–205.00)	**0.000**
	Granulocytes [10^6^/l]	100 (10–1,070)	40 (10–170)	230 (10–1,070)	**0.001**
	Leucocytes [10^6^/l]	9.81 (3.34–31.18)	7.01 (3.60–22.20)	15.09 (3.34–31.18)	**0.004**
	Monocytes [10^3^/l]	1.09 (0.24–12.80)	0.86 (0.24–1.90)	1.13 (0.26–12.80)	0.43
	IL6 [pg/ml]	33.53 (3.63–684.84)	10.97 (3.95–236.71)	83.42 (3.63–684.84)	**0.002**
	IL8 [pg/ml]	68.78 (5.67–752.26)	43.76 (15.54–752.26)	77.84 (7.87–1487.36)	0.46
Laboratory ascites	Protein [g/l]	10 (1–44)	9.5 (4.0–19.0)	10.0 (1.0–44.0)	0.65
	Granulocytes [10^6^/l]	48 (2–2,333)	48 (7–250)	48 (2–2,333)	0.97
	Leucocytes [10^3^/l]	256 (11–3,031)	467 (52–887)	218 (11–3,031)	0.40
	Lymphocytes/Monocytes [10^3^/l]	127 (5–740)	300 (39–740)	127 (5–698)	0.15
	IL6 [pg/ml]	642.67 (7.87–1487.36)	596.15 (109.56–1226.54)	653.58 (7.87–1487.36)	0.45
	IL8 [pg/ml]	83.75 (10.91–988.56)	44.91 (15.17–277.70)	95.55 (10.90–988.56)	0.15

*Data are medians and (ranges). The results of Mann–Whitney test for metric variables and Chi-square test for nominal variables comparing patients with low CRP profile to those with high CRP profile are shown with P–value (significantly different comparisons are marked in bold)*.

The median C-reactive protein (CRP) level of 48.8 mg/l in the blood were compatible with bacterial infections (upper limit of normal 5 mg/l), together with findings of SBP in 21% of the patients (Table 1). CRP performed well to detect SBP using receiver operator characteristic curve analysis (AUC = 0.901, *P* ≤ 0.001). In blood cultures, we detected members of the bacterial genera *Enterococcus, Escherichia, Sporosarcina*, and *Staphylococcus* in five patients. Additionally, we detected *Candida* spp. in blood culture of one patient. In ascites, we detected the genera *Enterococcus, Serratia*, and *Staphylococcus* in five patients and *Candida* spp. in one patient. None of the patients presented with cultural evidence of bacteria in both, blood and ascites.

There was also evidence of systemic inflammation with elevated levels of cytokines IL-6 (and IL-8, although not statistically significant), which are mainly produced by myeloid cells. The median levels assessed by ELISA assays were 33.53 and 68.78 pg/ml, respectively (Table 1). Reported mean levels in healthy individuals are 1.46 and 12.9 pg/ml, respectively, assessed by the same technique (25, 26). Ascites fluid showed higher median levels of both cytokines, with an almost 20-fold increase of median IL-6 levels in ascites compared to blood from the same patient.

### Differences in Patients With Systemic Inflammation

**Figure 1:**
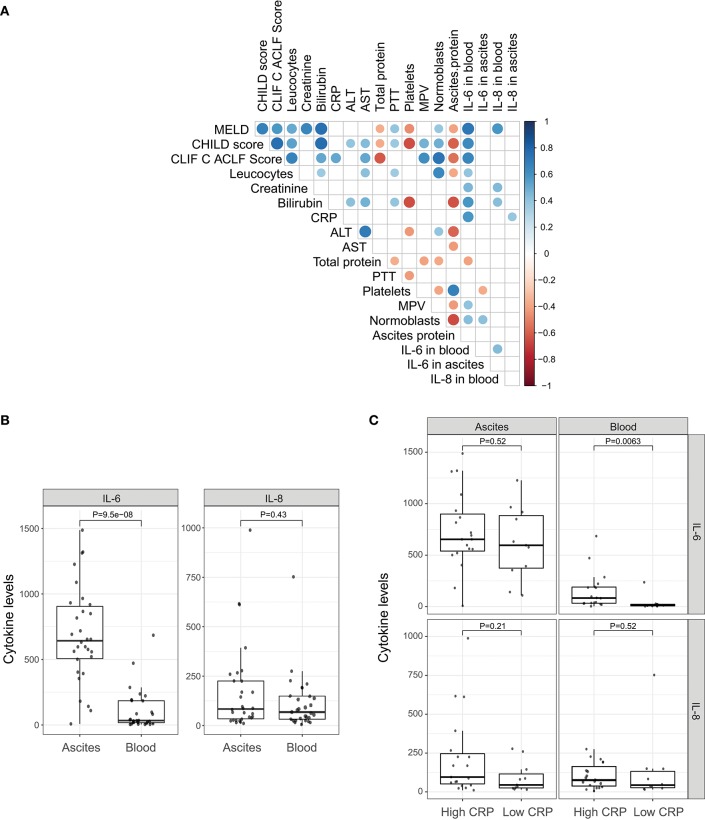
Pro-inflammatory cytokines. **(A)** Correlation of clinical markers for disease progression and systemic inflammation with cytokines IL-6 and IL-8 in blood and ascites. **(B)** Comparison between cytokines levels in ascites and peripheral blood. **(C)** Comparison between cytokines levels in ascites and peripheral blood under different systemic inflammation status (low CRP profile vs. high CRP profile).

In order to decipher the role of systemic inflammation, we divided the cohort into two groups according to their CRP level. A cut-off of 25 mg/l (sensitivity = 1.0, specificity = 0.54 for detection of SBP) was used to divide the cohort into 12 patients with a low CRP profile and 21 patients with a high CRP profile. Patients with the high CRP profile showed higher MELD, CHILD, and Chronic Liver Failure Consortium (CLIF-C)-ACLF scores (Table 1; *P* = 0.06, *P* = 0.03, and *P* = 0.002, respectively). SBP was detected in 50% of the patients (7 out of 14) with the high CRP profile, but in none of 12 patients with the low CRP profile (*P* = 0.02). Therefore, the positive predictive value, the probability for patients with high CRP profile to have SBP, is 35%. In contrast, the negative predictive value, the chance for patients with low CRP profile to not have SBP, is 100%. A high negative predictive value in this regards is extremely important since CRP level may be elevated also in absence of infection. Furthermore, patients with the high CRP profile showed a significant increase in blood granulocyte counts (*P* = 0.001), leucocyte counts (*P* = 0.004), and IL-6 levels (*P* = 0.002). However, there was no difference in ascitic cytokine levels between these two groups (Table 1). Similarly, IL-6 levels in blood strongly correlate with MELD, CHILD score, and CLIF-C-ACLF score and surrogate markers of systemic inflammation (leucocytes and CRP). However, blood IL-8 levels only slightly reflected disease stage and did not correlate significantly with markers of inflammation (Figure 1A). Even though median cytokine levels were higher in ascites than in blood (Figure 1B), ascitic cytokine levels reflected neither disease severity nor systemic inflammation (Figures 1A,C). Therefore, ascites cytokine levels did not seem to be affected by systemic inflammation or by the presence of SBP, which was diagnosed in one third of the patients with the high CRP profile (Figure 1C).

The rate of positive blood cultures was identical in patients with the low and high CRP profile. There were three positive blood and three positive ascites cultures in patients with a high CRP against two positive blood and one positive ascites cultures in the patients with lower CRP values.

### Characterizing the Microbiome in Ascites and Blood

We selected a subset of 10 representative patients (accounting for etiology and inflammation markers in ascites) from the whole cohort and performed 16S rRNA gene sequencing of ascites and blood to identify differences in microbial composition and diversity between ascites and blood. These 10 patients were also divided into two groups based on CRP levels (*n* = 4 for high CRP profile and *n* = 6 for low CRP profile, see Table 2). Their disease profiles with regard to stage of liver cirrhosis and inflammatory markers in blood were comparable to those of the respective subgroups and also of the whole cohort.

**Table 2:** Patient characteristics for the subpopulation in which 16S rRNA gene amplicon sequencing was performed in blood and ascites.

	**Parameters**	**Low CRP profile (*n* = 6)**	**High CRP profile (*n* = 4)**	***P***
General characteristics	Sex (male/female)	5/1	2/2	0.26
	Age, years	52.5 (40.0–67.0)	56.0 (33–76)	0.76
	Etiology of cirrhosis (alcohol/viral/other)	5/1/0	3/0/1	0.34
	MELD	10.26 (9.79–38.25)	18.72 (11.21–24.16)	0.17
	CHILD	9.0 (8.0–13.0)	9.5 (8.0–10.0)	0.91
	ACLF (no/yes)	4/2	1/3	0.20
	Esophageal varices (absent/small/large)	3/0/3	2/1/1	0.17
	Hepatic encephalopathy (absent/grade 1/grade 2/grade 3/grade 4)	5/1/0/0	3/1/0/0	0.34
	Hepatorenal syndrome (no/yes)	4/2	2/2	0.60
	Spontaneous bacterial peritonitis (no/yes)	6/0	3/1	0.20
	Blood culture (negative/positive/NA)	2/1/3	0/1/3	0.25
	Ascites culture (negative/positive/NA)	6/0/0	3/1/0	0.20
Laboratory blood	Creatinine [mg/dl]	0.98 (0.57–2.59)	2.27 (1.07–4.68)	0.17
	Platelets [10^6^/l]	69.5 (38.0–235.0)	191.5 (123.5–237.5)	0.26
	INR	1.35 (1.00–2.10)	1.20 (1.00–1.20)	0.47
	Bilirubin [mg/dl]	2.78 (0.75–45.21)	1.54 (0.73–7.97)	0.61
	GGT [U/l]	226.0 (152.0–585.0)	149.5 (78.0–1139.0)	0.47
	CRP [mg/l]	14.4 (5.7–22.2)	94.6 (27.6–205.0)	**0.01**
	Leucocytes [10^6^/l]	5.33 (3.60–9.07)	7.37 (6.59–17.30)	0.07
	Monocytes [10^3^/l]	0.57 (0.24–0.87)	1.10 (0.73–1.17)	0.10
	IL6 [pg/ml]	8.32 (3.95–236.71)	81.66 (19.94–97.29)	0.39
	IL8 [pg/ml]	41.80 (15.54–752.26)	66.61 (25.08–192.48)	0.61
Laboratory ascites	Protein [g/l]	8 (5–17)	21 (10–44)	0.11
	Granulocytes [10^6^/l]	18.5 (7–250)	24.0 (10.0–117.0)	0.91
	Leucocytes [10^3^/l]	130.5 (52.0–824.0)	163.0 (47.0–480.0)	0.61
	Lymphocytes/Monocytes [10^3^/l]	120.0 (39.0–740.0)	139.5 (37.0–363.0)	0.61
	IL6 [pg/ml]	757.19 (109.56–1226.54)	529.64 (7.87–816.83)	0.35
	IL8 [pg/ml]	29.93 (17.46–277.70)	116.82 (43.12–224.85)	0.17

*Data are medians and (ranges). The results of Mann–Whitney test for metric variables and Chi-square test for nominal variables comparing patients with low CRP profile to those with high CRP profile are shown with P-value (significantly different comparisons are marked in bold)*.

**Figure 2:**
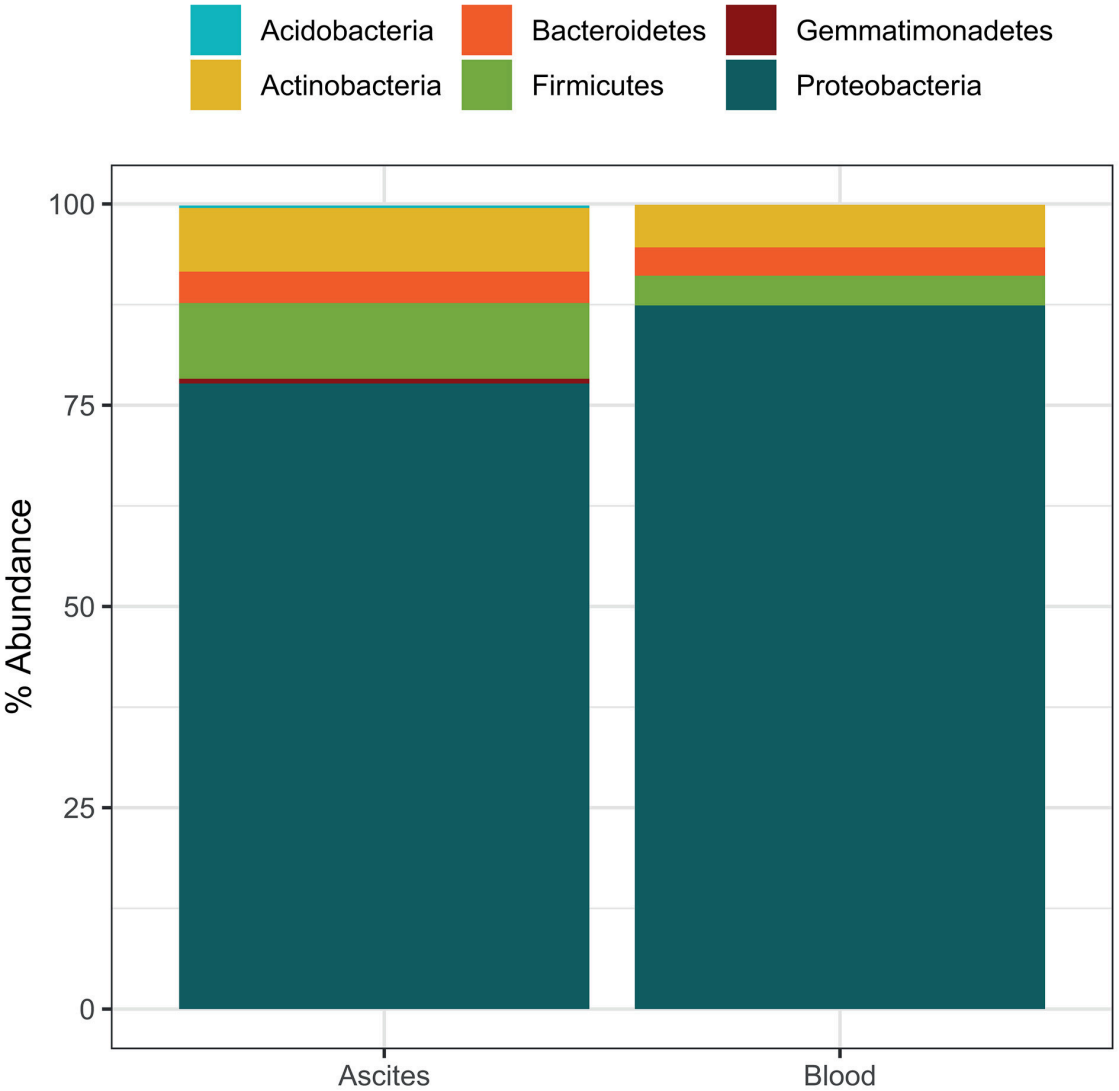
Average microbiome phylum composition in ascites and blood samples (*n* = 10 each).

Microbiota compositions at the phylum level were in line with previously reported bacterial compositions of ascites (27) and peripheral blood (12). The most abundant phylum detected in ascites samples was Proteobacteria (mean relative abundance 77.7%), followed by Firmicutes (9.4%), Actinobacteria (7.9%), Bacteroidetes (3.9%), and Gemmatimonadetes (0.6%) (Figure 2). The phylum composition was slightly different in the blood samples, with Proteobacteria accounting for 87.4% on average, while Actinobacteria, Firmicutes, and Bacteroidetes accounted for small proportions (5.3, 3.7, and 3.5, respectively; Figure 2). We identified 53 bacterial families in ascites and 48 families in blood samples. Among these, we found a set of six core families detected in all samples in both compartments: *Pseudomonadaceae, Oxalobacteriaceae, Neisseriaceae, Enterobacteriaceae, Sphingomonadaceae*, and *Moraxellaceae*. These results were in line with previous studies that reported an increase in potentially pathogenic families such as *Enterobacteriaceae* on the gut microbiome of liver patients and as well a high prevalence in ascites samples (28).

**Figure 3:**
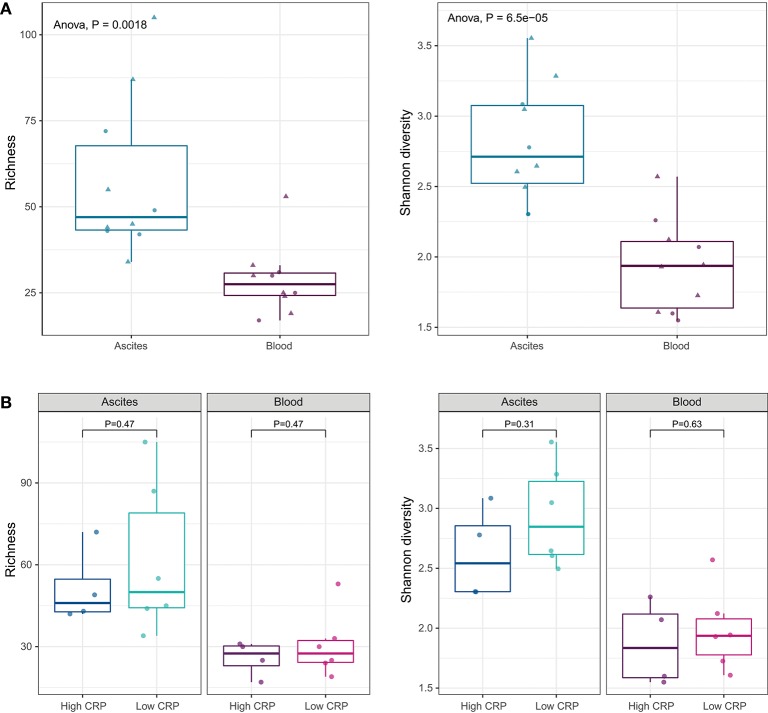
**(A)** Alpha diversity indices at genus level. Ascites microbiota has higher richness and diversity compared to peripheral blood microbiome. **(B)** Alpha diversity indices under different systemic inflammation status. The inflammation status does not affect the microbial diversity of the ascites or blood samples.

We identified 97 bacterial genera from ascites and 89 genera from blood (Figure S2). There were no statistical significant differences between the microbiota compositions of ascites and blood samples at phylum level. At genus level, alpha-diversity indices, such as richness (number of observed genera in a sample) and Shannon diversity (richness as well as evenness of genera in a sample), showed that the ascites microbiome was significantly more diverse than the peripheral blood microbiome (*P* < 0.01; Figure 3A).

Translocation of the gut microbiota into the circulatory system and into ascites is increasingly recognized as a driver of complication in advanced liver disease (27, 29). As we showed above, the levels of pro-inflammatory cytokines (IL-6 and IL-8) were higher in ascites compared to blood, suggesting different drivers of inflammation. To determine the relation of the microbiota with cytokine levels and systemic inflammation, we investigated the differences in the microbiome composition of ascites and blood samples, in patients with low and high CRP levels. CRP levels did not appear to affect microbiota diversity of ascites or blood samples (Figure 3B).

### Distinct Bacterial Signatures of Ascites and Blood Microbiome

**Figure 4:**
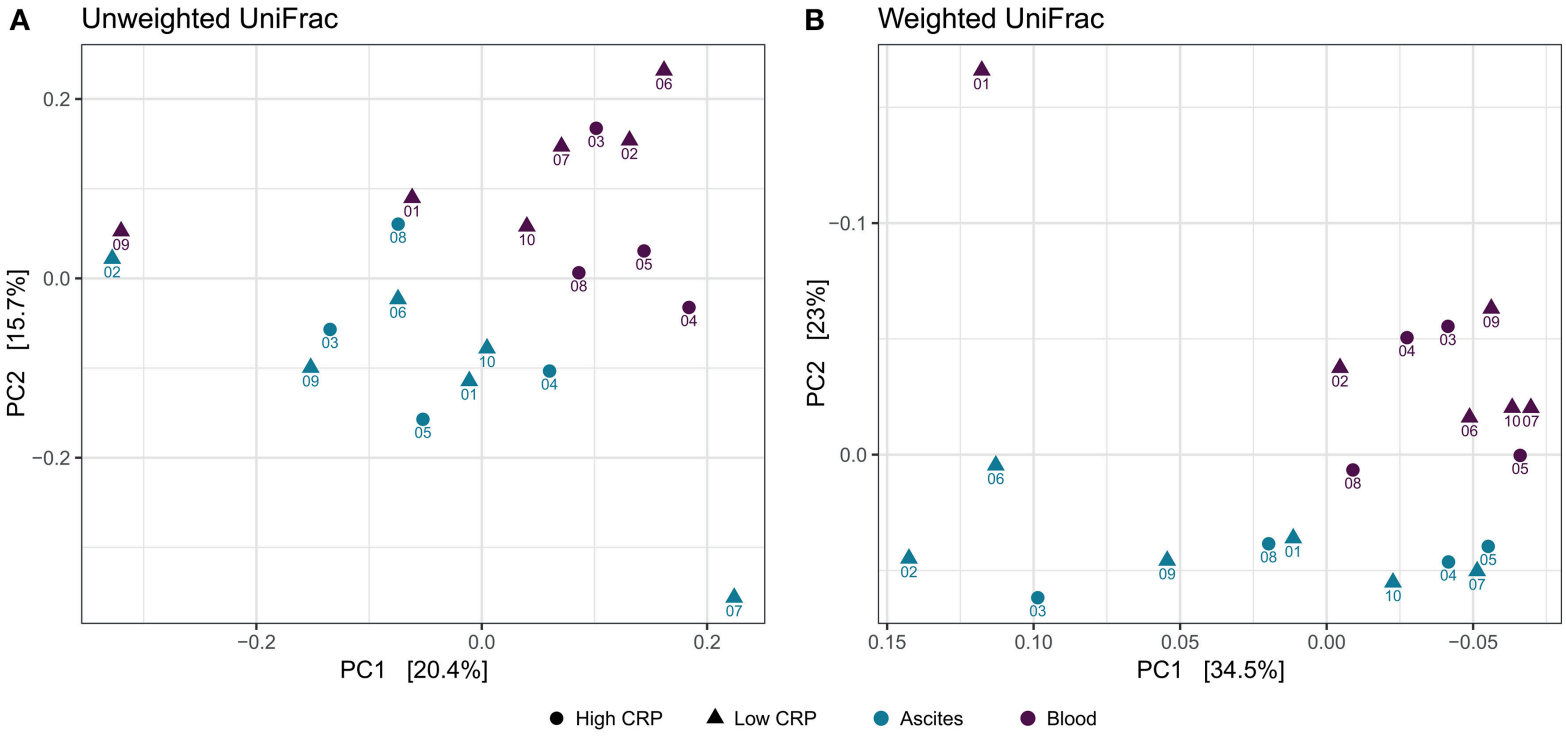
Beta-diversity analysis. Principal coordinate analysis shows clear separation between ascites and peripheral blood samples, but samples do not cluster based on inflammation status. **(A)** Unweighted UniFrac **(B)** Weighted UniFrac.

To study the variation in the microbial structure between ascites and peripheral blood samples, we used unweighted and weighted UniFrac metrics (30) as measures of the beta-diversity. Unweighted UniFrac uses a phylogenetic tree taking into account only the phylogenetic affiliation of members to evaluate the community structure and diversity, while weighted UniFrac additionally considers the relative abundance of the members of the community. Both measures clearly separated ascites from blood samples (Figure 4). The sampling compartment explained 11% of the variance in beta-diversity when using unweighted UniFrac metric (PERMANOVA test, *P* = 0.006), whereas it explained 21.2% of the variance when using weighted UniFrac metric (PERMANOVA test, *P* = 0.001). Additionally, the first principal coordinate (PC1) of microbiome beta-diversity explained 20.4% of the variance in the samples under unweighted UniFrac metric, whereas it explained 34% of the variance in the samples under weighted UniFrac metric (Figure 4).

**Figure 5:**
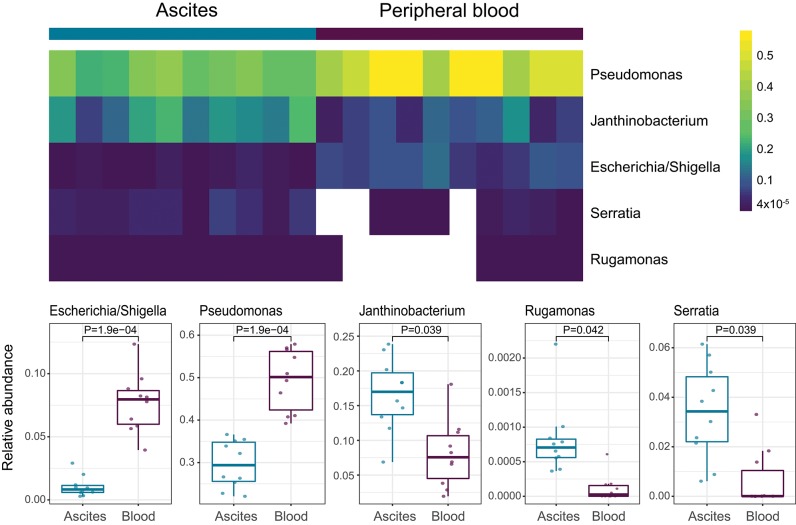
Genera differentially abundant among ascites and blood samples.

We then investigated specific bacterial signatures that differentiated ascites from peripheral blood samples. We used DAtest (24) to select the most suitable statistical method to identify differentially abundant bacteria. Compared with other statistical methods evaluated by DAtest, *t*-test had the highest power to distinguish between two simulated sample groups based on our cohort, while keeping the false discovery rate of simulated differential taxa under 0.05. Therefore, we performed *t*-tests between the ascites and blood microbiomes using the relative abundances of 99 genera. We found that *Janthinobacterium, Serratia*, and *Rugamonas* were significantly enriched in ascites, while *Escherichia/Shigella* and *Pseudomonas* were enriched in blood (Figure 5, Table 3). These findings were in concordance with our cultivation results, where *Serratia* was detected in ascites culture and *Escherichia* in peripheral blood.

**Table 3:** Differentially abundant genera between ascites and blood.

**Kingdom**	**Phylum**	**Class**	**Order**	**Family**	**Genus**	**Padj**	**Sig**.	**log2FC**	**Dir**
Bacteria	Proteobacteria	Gammaproteobacteria	Enterobacteriales	Enterobacteriaceae	Escherichia/Shigella	0.00019243	^***^	2.75	Blood
Bacteria	Proteobacteria	Gammaproteobacteria	Pseudomonadales	Pseudomonadaceae	Pseudomonas	0.00019243	^***^	0.74	Blood
Bacteria	Proteobacteria	Betaproteobacteria	Burkholderiales	Oxalobacteraceae	Janthinobacterium	0.038589035	^*^	−1.04	Ascites
Bacteria	Proteobacteria	Gammaproteobacteria	Enterobacteriales	Enterobacteriaceae	Serratia	0.038589035	^*^	−2.21	Ascites
Bacteria	Proteobacteria	Betaproteobacteria	Burkholderiales	Oxalobacteraceae	Rugamonas	0.041897934	^*^	−0.71	Ascites

Padj, adjusted P-value; Sig., significance level;

*** P < 0.001,

* *P < 0.05; Dir, direction of enrichment; log2FC, log2 of fold change*.

Since the presence of bacteria in blood is generally associated with systemic inflammation, we investigated whether there were differences in the bacterial composition of patients with systemic inflammation (high CRP levels, *n* = 4) and patients without systemic inflammation (low CRP levels, *n* = 6). There was no significant difference between groups, potentially due to limited sample size. We then examined the 5 bacterial genera that were significantly different between ascites and blood samples to determine their relationship with systemic inflammation. Although there were no statistically significant differences between the samples with and without systemic inflammation, *Escherichia/Shigella* and *Janthinobacterium* were more abundant in patients with elevated systemic inflammation, both in ascites and blood samples, while *Pseudomonas* and *Serratia* were increased only in the ascites samples with systemic inflammation (Figure S1).

## Discussion

The present study demonstrates that the levels of inflammatory cytokines and microbial richness are significantly higher in ascites of patients with decompensated cirrhosis than in their blood compartment. Neither the levels of cytokines nor the overall abundance of bacterial DNA nor microbial richness seemed to be related to the extent of systemic inflammation in these patients.

These findings are particularly striking, since the cell count in circulating blood is several fold higher than in ascites. Still, the levels of inflammatory cytokines were several fold reduced in blood compared to ascites. Moreover, the relationship between the surrogates of systemic inflammation (e.g., leucocyte count, CRP) and severity scores of liver cirrhosis were correlated with the levels of blood cytokines, but no relationship was detected in the same patients in ascites.

A recent study described the role of alcohol consumption for circulating microbiome composition and associated metabolic functions. Even though leucocyte count and endotoxemia increased with the severity of disease in this study, soluble CD14, as a marker for monocyte activation, decreased in severe alcoholic hepatitis underlining the dysregulation of the immune response (31).

Overall, these results may suggest a dysregulation in myeloid cell function in cirrhosis with an excess of cytokine synthesis in ascites. This does, however, not result in at least the two main markers of systemic inflammation investigated here. Although rather descriptive, our study suggests compartmentalization of the immune response in this setting. This compartmental dysregulation may occur as a response to MAMPs, e.g., bacterial DNA.

The reason for this compartmentalization of the immune response remains elusive. Granulocytes, as one of the main producers of cytokines, besides macrophages and monocytes, but crucial for the diagnosis of SBP, were similar or lower in ascites samples than blood samples, although levels of IL-6 and IL-8 were many times higher in ascites. This suggests an external source of these cytokines and inflammation in ascites. Bacterial translocation from the gut is suspected to be the primary source of infection in SBP (2). However, bacteria not associated to the gut can also be found in ascites, suggesting other additional sources of infection (27). Bacterial translocation has been associated with impaired immune response and decompensation of liver cirrhosis, but the efforts to elaborate simple biomarkers such as detection and cut-off levels of bacterial DNA have shown discrepant results. Such discrepancies highlight that the relationship of presence or amount of bacterial DNA is not a strong predictor for decompensation and systemic inflammation in these patients, and probably also does not explain compartmentalization of the immune response. However, recent studies show an association of bacterial DNA levels in ascites and survival (32, 33).

Therefore, we have characterized the microbiome of matched ascites and blood samples from patients with decompensated liver cirrhosis. While ascites and blood harbored the same phyla with marginally different proportions, they showed distinct compositions at genus level. Genus alpha-diversity represented by richness and Shannon's diversity was significantly higher in ascites than in blood. Depending on the choice of the beta-diversity measure, 11% (unweighted UniFrac), or 21.2% (weighted UniFrac) of the variance in beta-diversity was dependent on the sampling compartment. These results suggest that the phylogeny of the members as well as their relative abundance have a significant yet cumulative effect in explaining the differences between ascites and blood microbial communities.

Culture-positive organisms *per se* do not seem to trigger systemic inflammation assessed by two representative cytokines (9) in patients with cirrhosis as demonstrated by the blood and ascites culture results in the two sub cohorts stratified by their CRP profile. Therefore, we hypothesize that abundance of specific genera must play a prominent role in the induction of systemic inflammation in cirrhosis. This hypothesis is supported by other studies where specific bacterial species correlate with clinical measures (27, 31).

We also identified five bacterial genera that were significantly different between ascites and blood samples, despite the small cohort size. Some of these genera were also cultivated from additional samples of the respective compartments, confirming the viability of at least some of the bacteria reported in our study. Even though high CRP levels were associated with SBP, we did not see a significant difference in microbiome composition between high and low CRP samples, possibly due to reduced statistical power. However, the genera *Escherichia/Shigella* and *Janthinobacterium* showed higher abundance in blood samples of patients with systemic inflammation. *Escherichia* is already known for its pathogenic capabilities via lipopolysaccharides binding to Toll-like receptor 4, especially in individuals with dysfunctional immune response. *Janthinobacterium*, another Gram-negative bacterium, has not been described in relation to bacteremia previously, but could also induce inflammation via the same mechanism. On the other hand, while relative abundance of *Escherichia* was decreased in ascites samples, *Janthinobacterium* was increased. We speculate that the compartmentalization of immune dysfunction might be the reason behind the different behavior of these two bacteria, both of which are Gram-negative, in the different compartments.

In decompensated cirrhosis, representative markers of systemic inflammation and the microbiota seem to be dysregulated in ascites and blood. While a relationship between systemic inflammation and the microbiota seems to exist in blood, this is not found in ascites. These data may suggest the compartmentalization of the immune response and its interaction with the microbiome is especially important and evident in blood, while in ascites not found in our hands.

## Author Contributions

CA-S, RS, MA, and JT: conceptualization and methodology. CA-S, RS, AP, MA, and JT: formal analysis. CA-S, RS, AP, FM, FU, and PR: investigation. FM, FU, PR, and EL: resources. BL: data curation. CA-S, RS, EL, BL, JC, MV, MA, and JT: writing—original draft. CA-S, RS, MA, and JT: visualization. MA and JT: supervision.

### Conflict of Interest Statement

BL is an employee of Vaiomer. MV has served at the speakers' bureau of Akademie für Infektionsmedizin, Ärztekammer Nordrhein, Astellas Pharma, Basilea, Gilead Sciences, Merck/MSD, Organobalance, and Pfizer, received research funding from 3M, Astellas Pharma, DaVolterra, Evonik, Gilead Sciences, Glycom, MaaT Pharma, Merck/MSD, Morphochem, Organobalance, Seres Therapeutics, Uniklinik Freiburg/Kongress und Kommunikation and is a consultant to Alb-Fils Kliniken GmbH, Ardeypharm, Astellas Pharma, Berlin Chemie, DaVolterra, Ferring, MaaT Pharma, and Merck/MSD. JT has served as speaker or consultant for Gore, Bayer, Alexion, MSD, Gilead, Intercept, Norgine, Grifols, Versantis, and Martin Pharmaceutical. The remaining authors declare that the research was conducted in the absence of any commercial or financial relationships that could be construed as a potential conflict of interest.
