# Supplementary material for: Compartmentalization of Immune Response and Microbial Translocation in Decompensated Cirrhosis
Source: Front Immunol. 2019 Feb 8;10:69. doi: 10.3389/fimmu.2019.00069 (PMC6376951; doi:10.3389/fimmu.2019.00069)
Supplement: Supplementary file 1 [file Image_1.pdf]

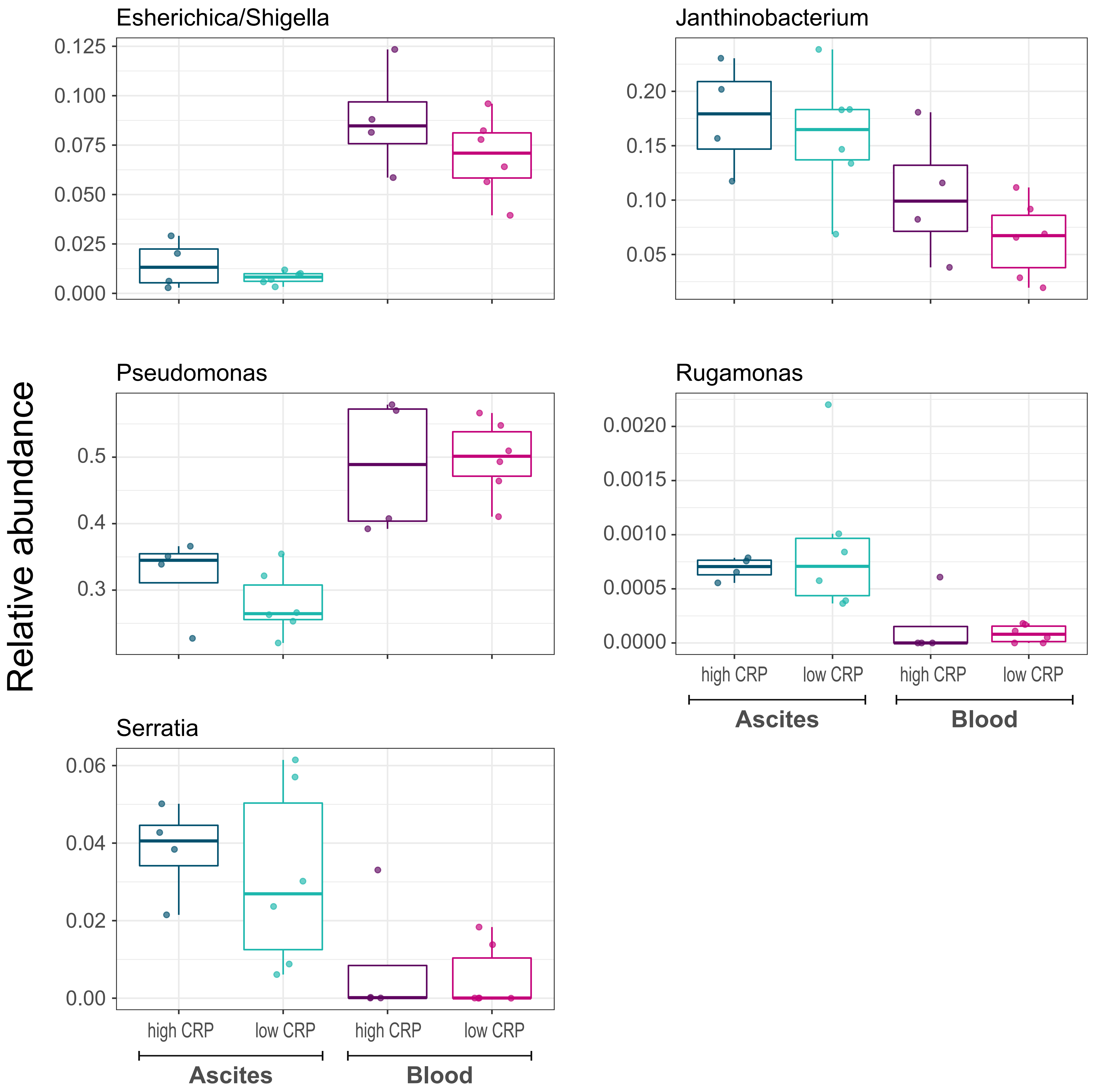

Figure S1. Genera differentially abundant between patients with high CRP and low CRP. Comparisons were made separately for ascites and blood samples.
