# Supplementary material for: Compartmentalization of Immune Response and Microbial Translocation in Decompensated Cirrhosis
Source: Front Immunol. 2019 Feb 8;10:69. doi: 10.3389/fimmu.2019.00069 (PMC6376951; doi:10.3389/fimmu.2019.00069)
Supplement: Supplementary file 2 [file Image_2.pdf]

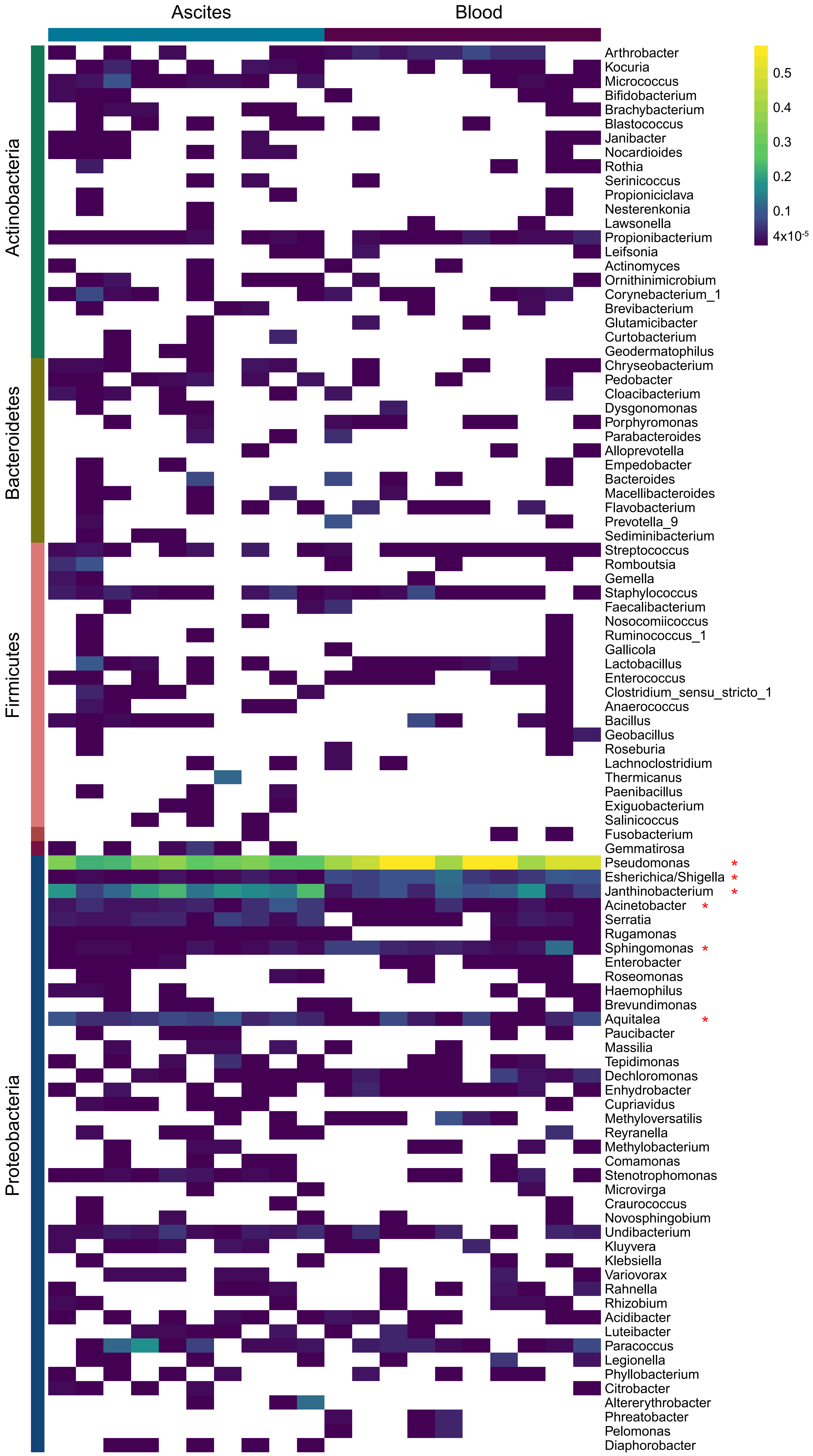


**Figure S2.** Microbiome composition in our cohort of 10 patients. Heatmap shows the relative abundance of different genera. Phylum affiliation of genera are marked to the left. Six core genera detected in all samples in both compartments are marked with red asterisks.
